# Physical Aspects of Organogelation: A Point of View

**DOI:** 10.3390/gels7020065

**Published:** 2021-06-01

**Authors:** Jean-Michel Guenet

**Affiliations:** Institut Charles Sadron, CNRS—Université de Strasbourg, 23 rue du Loess, CEDEX 02, F-67034 Strasbourg, France; jean-michel.guenet@ics-cnrs.unistra.fr; Tel.: +33-388-41-4000

**Keywords:** organogel, phase diagram, morphology, molecular structure, rheology

## Abstract

The physics side of organogelation is broached through three main aspects, thermodynamics (formation and melting), structure (morphology and molecular organization), and rheology. A definition of a gel is first discussed so as to delimit the field of investigation; namely, systems constituted of fibril-like entities. It is again highlighted that gel formation occurs through first-order transitions, chiefly by homogeneous nucleation. A deeper knowledge of the system is thus achieved by mapping out the temperature–concentration phase diagram. Some experimental diagrams are shown, while diagrams likely to pertain to these systems are presented. The molecular arrangement is basically crystallization that occurs in a preferred direction, hence the formation of fibrils. The effects of the solvent type, the quenching process of the solution are discussed with respect to the morphology and the crystal structure. Finally, the rheological properties are tackled. Notions of critical gelation concentration and percolation are debated. The interest of mapping out the temperature–concentration phase diagram is emphasized, particularly for understanding the variation of the gel modulus with temperature.

## 1. Introduction

Organogelation is a relatively-new topic that has emerged these past 20 years [1,2,3,4,5]. From a few papers in the early nineties, the number of papers amounts now to more than 200/year when one enters in databases the single keywords “organogelation”, or “organogel” [6]. So far, most of the literature deals with the synthesis of organogelators (also named low molecular weight gelators as opposed to covalent polymers) together with a basic characterization. What are organogels, how and why do they form, what are the relations between their morphology/structure with their properties, particularly with their rheological properties? This special issue is an opportunity for discussing some important physical aspects that should be addressed in order to gain a deeper understanding of these systems. In this aim, some basic principles are presented and basic investigations are suggested. Some recent experimental results illustrate the physics-oriented approach of this topic, yet this article is by no means a comprehensive review on organogels.

## 2. Results and Discussion

The first section will be devoted to defining what can be termed organogelation and what are the objects associated with this concept. The thermodynamics of organogel formation is an important step in understanding these systems, particularly through the mapping out of the temperature-composition phase diagram. The different types of morphologies and molecular structure will be then examined. A final section will be devoted to their rheological properties.

### 2.1. A Definition

The purpose of a definition is to specify the extension of a concept, namely, here, organogelation, and to identify the objects belonging to a specific set, namely, objects possessing common characteristics and/or properties. Most books and papers attempting to define a gel quote the famous sentence by Dorothy Jordan Lloyd: “*The colloidal condition, the gel, is one which is easier to recognize than to define*” [7]. This question has been largely tackled in the case of polymer thermoreversible gels with always the same ambiguous answers [8,9,10,11]. To be sure, a gel is capable of “jailing” a large amount of solvent, the latter being by far the major component, which is probably the only unquestionable statement. In principle, a gel is supposed to behave as a solid material that possesses an elastic modulus at zero frequency in oscillatory measurements or at infinite time in relaxation experiments. Most of polymer thermoreversible gels are not endowed with this property, and yet their gel status is usually not disputed [10]. Similar observations have been recently reported for organogels [12].

Guenet has suggested replacing the rheological definition, which is too restrictive, by contemplating two criteria that involve the topology of the gel and its thermodynamic property [5,11]. As commonly accepted, a gel is basically a network, whose definition as given in any language dictionary is: “a large system of lines, tubes, wires, etc. that cross one another or are connected with one another”. This lays the ground for the topological criterion. As these gels are thermoreversible, namely, they melt on heating and reform on cooling for unlimited cycles, the thermodynamic criterion states that the gel formation/melting process must occur through first order transition. These two criteria define a set of objects that can be designated as fibrillar organogels [5,11].

**Figure 1:**
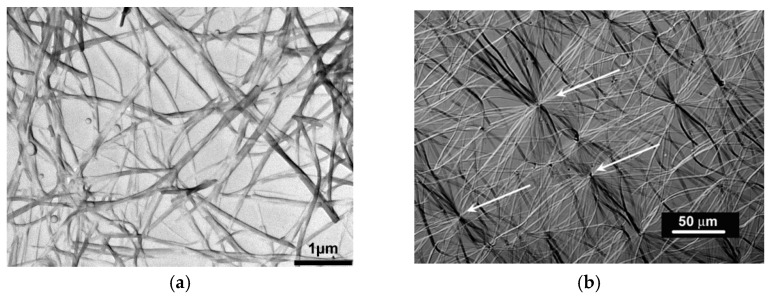
(**a**) TEM micrograph of an OPVR/*trans*-decahydronaphthalene, which shows a typical randomly-dispersed network (**b**) optical micrograph of an OPVOH/*trans*-decahydronaphthalene organogel displaying a hub-like network where fibrils radiate from and connect to nodes (arrows). Chemical structures in Appendix A Figure A1 [13,14].

Figure 1 shows two examples of organogels complying with the first criterion as they are made up with fibrils connected randomly (randomly-dispersed network) or connecting at a center (hub-like network) [13,14]. As will be shown below, these gels do form and melt through first order transitions. We shall see in the section devoted to rheology that some fibrilllar organogels obey the rheological definition, some obey it partially, and some do not at all. The fibrillary nature has generally an impact upon the visual aspect: a gel is usually slightly translucent, even transparent in some cases, with a blueish tinge due to blue light being more scattered than the other radiations (see 15 for details). Usually, when a system displays strong turbidity its gel status is rarely confirmed.

Before proceeding further a short comment on an all-too-often used test, namely, the tilting tube method, is worth bringing up. This test, which consists in determining the jamming of the solution, is strongly deceiving as many systems that would not be considered a gel would pass the test. For instance, no flow can be observed after the formation of an array of spherulites, which is by no means a gel according to the above definition [5,15,16].

### 2.2. Thermodynamics

The thermodynamics of organogelation will be approached through two aspects: the fibril growth mechanism and the mapping out of the temperature-composition phase diagram.

#### 2.2.1. Fibrils Growth

**Figure 2:**
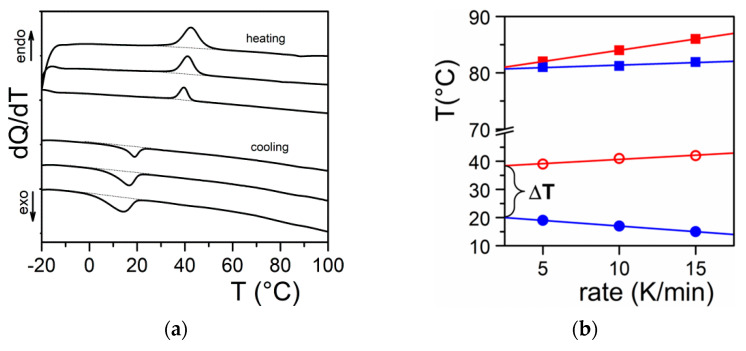
(**a**) DSC thermograms obtained on heating (endo) and on cooling (exo). Scanning rates 5, 10, and 15 °C/min; (**b**) variation of the melting peak peak (**◯**) and the formation peak (●) for Tri-aryl-triamine/tetrachloroethane organogel (C = 0.02 g/g). (■) and (■) stand for pure naphthalene for the sake of comparison.

**Figure 3:**
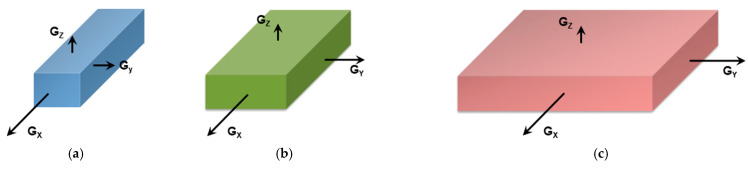
(**a**) fibrillary shape due to the fact that the growth rate along the x-direction, G_x_, is much faster than those in the other two directions (G_x_ >> G_y_ and G_x_ >> G_z_). (**b**) formation of a lath when G_x_ > G_y_ >> G_z_; (**c**) when G_x_ = G_y_ >> G_z_, then formation of extended platelets is expected.

It should be realized that organogelation is chiefly a crystallization process as we shall discuss in more detail below. This is therefore a *nucleation-controlled* phenomenon [17,18,19]. DSC experiments do highlight this process through the observation of formation exotherms and melting endotherms (Figure 2a). That fibrillar structures are obtained arises simply from the faster growth of one crystal face with respect to the other two as highlighted in Figure 3. Here, for fibrils to grow one has G_x_ >> G_y_ and G_x_ >> G_z_ (Figure 3a). If G_x_ >> G_z_ with G_x_ > G_y_ (Figure 3b) one may expect the formation of lathes. Finally, if G_x_ ≈ G_y_ >> G_z_ (Figure 3c), then platelets and correspondingly spherulites are obtained. The resulting spherulitic morphology does not comply with the topological criterion discussed above.

**Figure A1:**
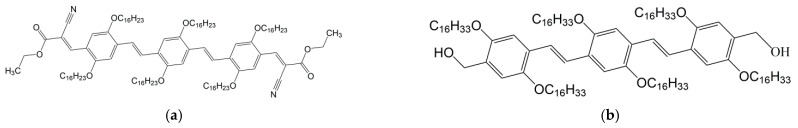
(**a**) OPVR; (**b**) OPVOH. For synthesis see reference [4].

Why is there growth in a privileged direction? In a recent monograph Guenet regards the low-molecular-weight molecules involved in organogelation as *chimeras*. Indeed, the organogelators display differing facets as do these creatures of the Greek mythology [5]. A typical example is given by oligo phenylene vinylene molecules (OPV) studied by Ajayaghosh and coworkers [4] (see Appendix A Figure A1). One growth face involves π-stacking, the second one H-bonding, and the third one van der Waals interactions. In this system the fastest growth occurs through the π-π interactions. The *chimera* character of the organogelators has another probable consequence on the onset of the gelation process. As highlighted in Figure 2b one usually observes a large undercooling Δ*T* between the formation temperature and the melting temperature. This is typical of a *homogeneous nucleation* phenomenon as opposed to a *heterogeneous nucleation* process that usually occurs for simple molecules such as naphthalene as also shown in Figure 2b. Gibbs has derived the following expression relating Δ*T* to the value of the critical radius ρ_c_ necessary for triggering the crystal growth [20]:

(1) $$\rho_{c}=\frac{\sigma T_{m}^{o}}{\Delta H_{m}\Delta T},$$

where *σ* is the surface free energy, Δ*H_m_* the melting enthalpy, and $T_{m}^{o}$ the equilibrium melting temperature of the infinite crystal. This clearly shows that the higher Δ*T* the lower the critical size necessary for triggering the growth process.

Also, the number of nuclei N depends on Δ*T* through [19]:

(2) $$N\sim \;exp-\left[\frac{16\pi \sigma^{3}{\left(T_{m}^{o}\right)}^{2}}{3kT\Delta T^{2}\Delta H_{m}^{2}}\right],$$

This equation implies a rapid increase of the number of nuclei with increasing Δ*T*, and correspondingly a rapid decrease of the size of the crystals/fibrils. This will be further illustrated in the section devoted to molecular structure.

Alternatively, *heterogeneous nucleation* needs only any impurity displaying adequate interaction with the molecule for reaching rapidly the critical nucleus size. Small molecules are more likely to encounter such an impurity unlike complex molecules such as organogelators. Therefore, only self-seeding is likely to be at play in the formation of most of the organogels.

Another consequence of the nucleation-controlled process implies that gel formation cannot be treated as a chemical equilibrium. Owing to the necessary undercooling for reforming the gel after melting, gelation is not a strictly reversible process unlike chemical equilibrium or second order transitions. Accounting for the thermodynamic properties, particularly the melting enthalpy, by assuming a chemical equilibrium is utterly misleading. In many papers the van’t Hoff, Le Chatelier and Schröder relation is abusively used [21]:

(3) $$Ln\;X=-\frac{\Delta H_{m}}{R}\left[\frac{1}{T\left(X\right)}-\frac{1}{T_{m}}\right],$$

where *X* is the organogelator composition, Δ*H_m_* the associated enthalpy, *T_m_* and *T*(*X*) the melting temperatures of the pure gelling component and the component at composition *X*, respectively.

This may work for ideal solutions, which is highly improbable with organogelators. Misuse of this equation was unwittingly demonstrated by Shinkai and coworkers [22]. The enthalpies they derived from the variation of *T*(*X*) vs. *X* exhibit large discrepancies with regard to those obtained from DSC that are beyond experimental uncertainties.

Feng and Cavicchi [23] were perfectly aware of the irrelevancy of Equation (3), and suggested introducing a solvent/organogelator interaction parameter *χ_int_* for a more realistic approach:

(4) $$\ln X+{\left(1-X\right)}^{2}\chi_{int}=\frac{\Delta H_{m}}{R}\left(\frac{1}{T_{m}}-\frac{1}{T\left(X\right)}\right),$$

Relation (4) can pertain to the simplest case of a solid–liquid transformation, it cannot definitely account for more complex situations as will be detailed in the next section, particularly when liquid–liquid phase separation come into play or when molecular compounds are formed.

#### 2.2.2. Phase Diagrams

The mapping out of the temperature-composition phase diagram is an important step in the study of organogels. It should be carried out first as it delivers essential pieces of information for the determination of the structure, and properties of the gels. A few theoretical cases are presented in what follows together with some experimental examples. To be sure, the experimental phase diagram can be a combination of these typical cases.

These temperature-composition phase diagrams are constructed by applying the two fundamental Gibbs’phase rules [20,24,25]:

- the *variance* of the system *v*, which stands for the number of variables that can be changed without altering the state of the systems. When only the temperature is varied (all other external stimuli being kept constant), the variance reads:
  (5) v=N−φ+1,
  where *N* is the number of components and *φ* the number of phases. For two components, the number of phases cannot exceed *φ* = 3 as the variance is *v* = 0 under these conditions. This implies that the co-existence of the three phases is restricted to a point. The notion of variance possesses another key outcome, the occurrence of *non-variant thermal events*, namely, the transition temperature remains constant in large range of composition. Note that the composition is always given in *w/w* or mol/mol so that it does not depend upon temperature, while the concentration expressed in g/cm^3^ does.
- the *lever rule* which allows one to calculated the different proportions of the phases.

It is worth emphasizing that only the melting temperature is a thermodynamic characteristic unlike the formation temperature, as the latter is nucleation-controlled, and therefore depends upon external factors. Yet, the melting temperature depends upon the size of the crystals. The Gibbs-Thomson equation (actually first derived by Rie) [26] relates the melting temperature *T_m_* of finite-sized crystals to that of infinitely-large crystals $T_{m}^{o}$ through:

(6) $$T_{m}=T_{m}^{o}\left[1-\frac{S\sigma }{V\Delta H_{m}}\right],$$

where *V* is the crystal’s volume, *σ* and Δ*H_m_* the same as in relation 1. For cylinders of cross-section *r*, relation 6 reduces to

(7) $$T_{m}=T_{m}^{o}\left[1-\frac{2\sigma }{r\Delta H_{m}}\right],$$

And for rectangular cross-section of length *l_a_* and width *l_b_*:

(8) $$T_{m}=T_{m}^{o}\left[1-\frac{2\left(l_{a}+l_{b}\right)\sigma }{l_{a}l_{b}\Delta H_{m}}\right],$$

In most cases, cross-sections are large enough so that the effect is rather limited, or even undetectable as far as the melting temperature is concerned. Yet, this is something to keep in mind when processing the DSC data; the more so as fibrils cross-sections depend on the depth of the quench (see section devoted to molecular structure).

1. Solid–liquid transformation

**Figure 4:**
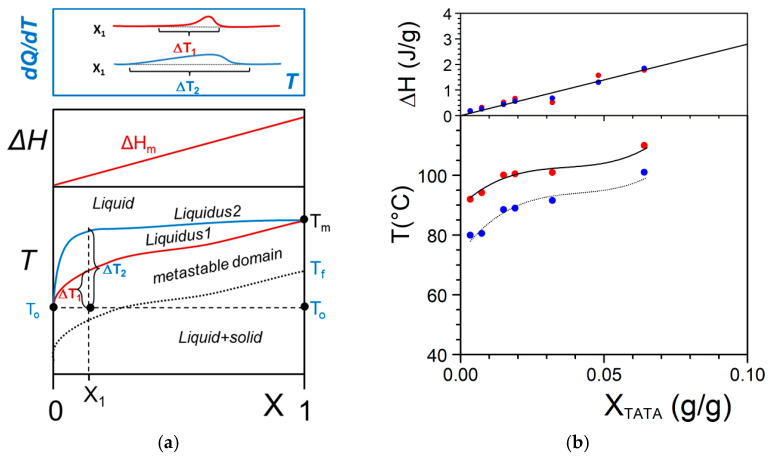
(**a**) The simplest T-X phase diagram for a two-component system. Two liquidus lines are considered with two different Δ*T* at a given [*X*_1_,*T*_o_] coordinate. The dotted black line stands for the gel formation. *T*_m_ and *T*_f_ are the melting and the formation temperatures of the pure gelator. Top, Tamman’s diagram. Above: shape of the expected endotherms; (**b**) experimental T-C phase diagram for Tri-aryl triamine/bromobenzene organogels (chemical structure in Appendix A Figure A2) [27,28]; (●) = melting, (●) = formation. Note the low composition range for the gelator.

The simplest case is shown in Figure 4a where the melting temperature of the gel, defined as the liquidus line, increases monotonously with the gelator composition. On cooling two phases form: the *gelator-poor phase* (the dilute phase), and the *gelator-rich phase* (the gel). At the liquidus line the following transformation occurs:

(9) liq+solid →liquid,

The proportion of the different phases is given by the lever rule. For example the fraction *φ* of the different phases read at a temperature *T*:

(10) $$\varphi_{poor}=\frac{X_{rich}\left(T\right)-X\left(T\right)}{X_{rich}\left(T\right)-X_{poor}\left(T\right)}\;\mathrm{and}\;\varphi_{rich}=\frac{X\left(T\right)-X_{poor}\left(T\right)}{X_{rich}\left(T\right)-X_{poor}\left(T\right)}\;,$$

where *X*(*T*), *X_poor_*(*T*) and *X_rich_*(*T*) are the compositions of the solution, the poor phase and the rich phase, respectively, at a temperature *T*. For example sample standing at *T* = T_o_ for X_1_ one has *X_poor_*(T_o_) ≈ 0 and *X_rich_*(T_o_) = 1, and therefore *φ_poor_* = 1− X_1_ and *φ_rich_* = X_1_.

The increase of the liquidus line in the low-composition domain can be gradual or abrupt. Consequently the dilute phase composition can be almost zero or still contain some isolated gelator molecules above T_o_ (see Figure 4a). The shape of the liquidus has also a direct bearing upon the spreading of the melting endotherm as shown in Figure 4b. For a given composition and temperature, the distance to the liquidus, Δ*T_n_*, is significantly larger for the abrupt case. In any case, the melting enthalpy, Δ*H_m_*, must increase linearly from 0 to the melting enthalpy of the pure component (Figure 4a).

It is worth emphasizing that there exists a metastable domain observed on cooling due to the nucleation-controlled gelation process (see again Figure 2b) [29]. In principle the gelation line should simply be a shift of the liquidus line as is highlighted in Figure 4a. 

In Figure 4b are plotted the date collected by DSC on Tri-aryl triamine/bromobenzene organogels. These results illustrate perfectly the theoretical case displayed in Figure 4a. It is further shown that the enthalpies associated with the gel formation and the gel melting obtained at finite rates are the same or nearly the same. This outcome demonstrates the absence of a kinetic effect. Should this effect occur, then the formation enthalpy would be smaller than the melting enthalpy. Therefore, gel formation is virtually instantaneous here, as is in most cases.

2. Liquid–Liquid phase separation prior to gelation

**Figure 5:**
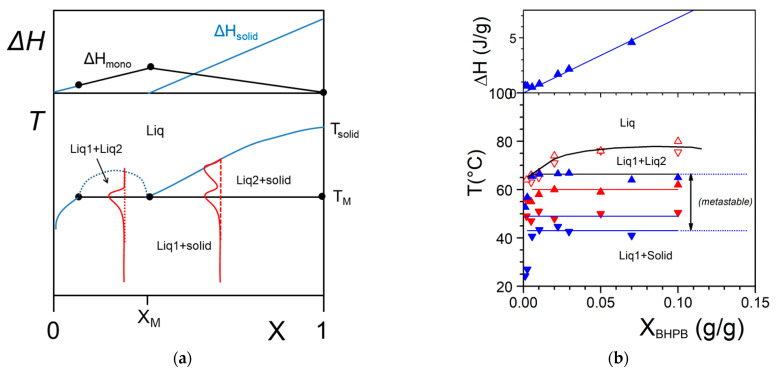
(**a**) A T-C phase where a monotectic transformation is observed. At the monotectic point the solid transforms into two liquids of differing compositions. The red lines show the shapes endotherms expected by DSC. The variations of the enthalpies associated with the different transitions are shown above. (**b**) Experimental example observed with BHPB-10 in *trans*-decahydronaphthalene (chemical structure in Appendix A Figure A3), X_BHPB_ is in gram of organogelator per gram of gel. The red arrows stand for optical microscopy investigations, solid symbols = formation and melting of the gel, open symbols = liquid–liquid phase separation; blue arrows stand for the DSC data. Arrow orientation indicates cooling (down) or heating experiments (up); The slight discrepancy between DSC temperatures and optical microscopy findings arises from the use of different cooling and heating rates (5 °C/min and 0.5 °C/min); above Tamman’s diagram [30].

As aforementioned, more complex systems may occur. The case of a liquid–liquid phase separation process that interferes with gelation has already been observed [30]. A typical phase diagram is shown in Figure 5a. The binodal line defines the miscibility gap where two liquids coexist. This type of transformation is designated as a *monotectic transition*. At the monotectic point X_M_, one obtains the following transformation:

(11) solid+liquid1 →liquid2,

By cooling within the miscibility gap (dotted line in Figure 5a) the solution decomposes first into two liquids prior to crystallizing. Crystallization eventually occurs as soon as the monotectic line is crossed. As a result, the outcome, and correspondingly the gel morphology, can differ whether the system consists of solution prepared below or above the monotectic composition X_M_. Below X_M_ the final gel morphology is decided by the first-occurring liquid–liquid phase separation (except for a small region at very dilute compositions). Above X_M_, a simple crystallization occurs which is followed by a change in liquid composition below T_M_. Here, the use of relations 3 and 4 is totally irrelevant as the gel melting remains constant in a large range of composition.

The system BHPB-10 in *trans*-decahydronaphthalene illustrates this type of situation (Figure 5b) where one observes a miscibility gap together with a non-variant event. There also exists a significant metastable domain revealed both by DSC and optical microscopy [30].

**Figure 6:**
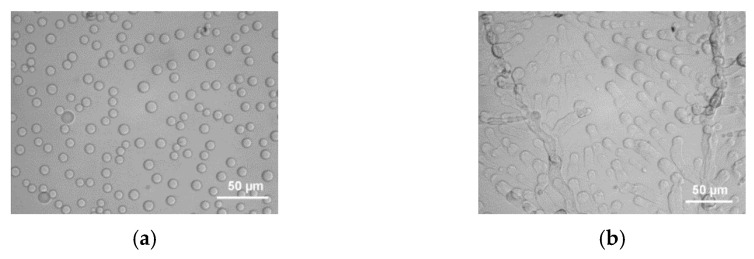
Optical micrographs obtained on BHPB-10/*trans*-decahydronaphthalene systems X = 0.05 g/cm^3^ (**a**) within the miscibility gap; (**b**) just after crossing the monotectic line [30].

The occurrence of a liquid–liquid phase separation is backed up by optical microscopy where droplets are seen prior to gelation (Figure 6a). Then, gelation takes over by forming snake-like structures that connect the droplets (Figure 6b). The aspect of the gel depends considerably upon the cooling rate for X < X_M_. Cooling slowly allows the growth of droplets before gelation sets in. Conversely, a rapid cooling allows the system to cross the monotectic line before decomposition into two liquid phases can take place. Under these conditions, no special features can be observed in optical microscopy (Figure 6). It will be seen in the next section that the gel morphology is totally different. Yet, the way the gel is prepared, slow cooling or rapid quench, has no effect on the melting properties as ascertained by DSC.

3. Molecular compounds organogelator/solvent

**Figure 7:**
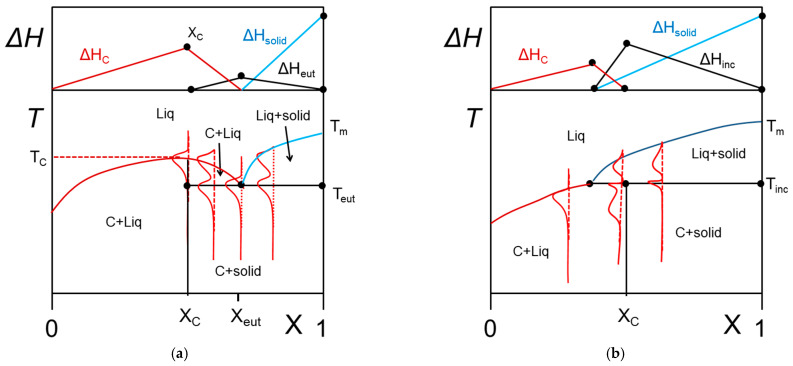
T-C phase diagrams. The red lines show the corresponding DSC traces. The Tamman’s diagram for the enthalpies associated with the different thermal events is shown on top. (**a**) for a molecular compound formed at X = X_c_. The complex further forms an eutectic compound at X = X_eut_. (**b**) an incongruently-melting compound where the molecular compound decomposes into a liquid phase and a solid phase at its stoichiometric composition.

The occurrence of molecular compounds that form between the organogelator and the solvent has already been reported in several papers [14,31,32]. To the best of the author’s knowledge no extensive phase diagrams have been mapped out for these systems. Theoretical examples of what would be expected with these systems are displayed in Figure 7.

In Figure 7a a *congruently-melting compound* is considered. This means that the compound has a well-defined stoichiometry at X = X_C_ (number of solvent molecules/gelator molecules), and behaves as a pure substance since it possesses its own melting point at T = T_C_ at its stoichiometric composition. Usually, the molecular compound is likely to form an eutectic compound with the solid phase beyond X_C_. At the stoichiometric concentration X_C_ one therefore observes the following transformation:

(12) C→liquid,

In Figure 7b, the phase diagram for an incongruently-melting compound is represented. While this type of compound possesses also a well-defined stoichiometry, it transforms into a solid phase prior to melting for compositions below the stoichiometric composition. At X_C,_ the following reaction occurs:

(13) C→solid+liquid,

The transformation at T_inc_ is also designated as a peritectic transformation.

From the Tamman’s diagram, namely, the variation of the enthalpies associated with the different transformations, one can easily guess whether one is dealing with a compound and its nature (congruent or incongruent). Admittedly, the phase diagram of Figure 7 may be confused with the simple case shown in Figure 4 in the low concentration range. As long as larger concentrations are not investigated definite conclusions cannot be drawn. Yet, for many reasons, such as synthesizing large amounts of sample, reaching large organogelator concentrations may not be possible. Conversely, the value of the melting enthalpy can convey some hints as to whether a compound is involved or not. By extrapolation of the data in the low concentration range to X = 1, one should retrieve the value of Δ*H_m_* of the pure crystalline state of the organogelator. Dasgupta et al. have reported in the case of OPVOH/benzyl alcohol gels that the extrapolated value of ΔH is larger by about 30% (240 J/mol against 180 J/mol) [14]. This clearly points to the existence of a molecular compound between OPVOH and benzyl alcohol, possibly formed through hydrogen bonds between the OH groups.

4. Metatectic tranformation

**Figure 8:**
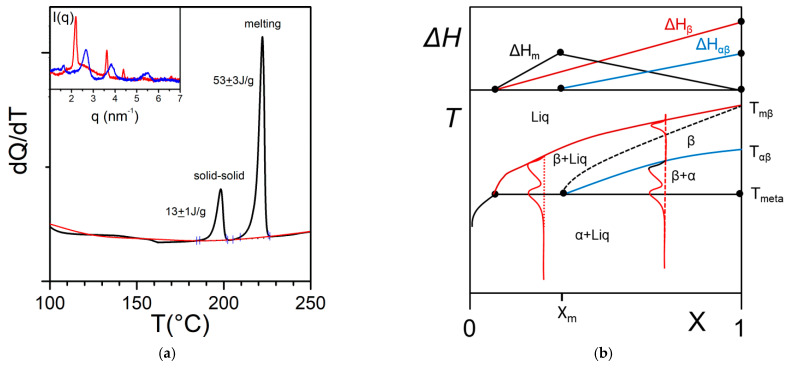
(**a**) The metatectic transformation can exist if there exists two crystalline forms, α form and β form of the gelator in the solid phase (here a tri-aryl tri-amine, TATA, shown in Appendix A Figure A2). At T_αβ_ = 198 °C the α form transforms into the β form as evidenced by the change of diffraction pattern in the inset: blue line at T = 20 °C, red line at T = 200 °C. (**b**) T-C phase diagrams for a metatectic transformation. The red lines show the corresponding DSC traces. The Tamman’s diagram for the enthalpies associated with the different thermal events is shown on top.

In some cases, the organogelator can exhibit two crystalline forms in the solid state, and a α form that transforms into a β form at T_αβ_ (solid-solid transformation Figure 8a). At the metatectic point one obtains:

(14) α+liquid→β,

The gel state is then likely to exhibit a metatectic transformation as detailed in Figure 8b. The observation of three melting endotherms for X > X_m_ is the signature of this transformation. The metatectic composition X_m_ may be relatively large so that not observable in usual gel investigations where only low concentrations are involved.

### 2.3. Morphology, Molecular Structure

**Figure 9:**
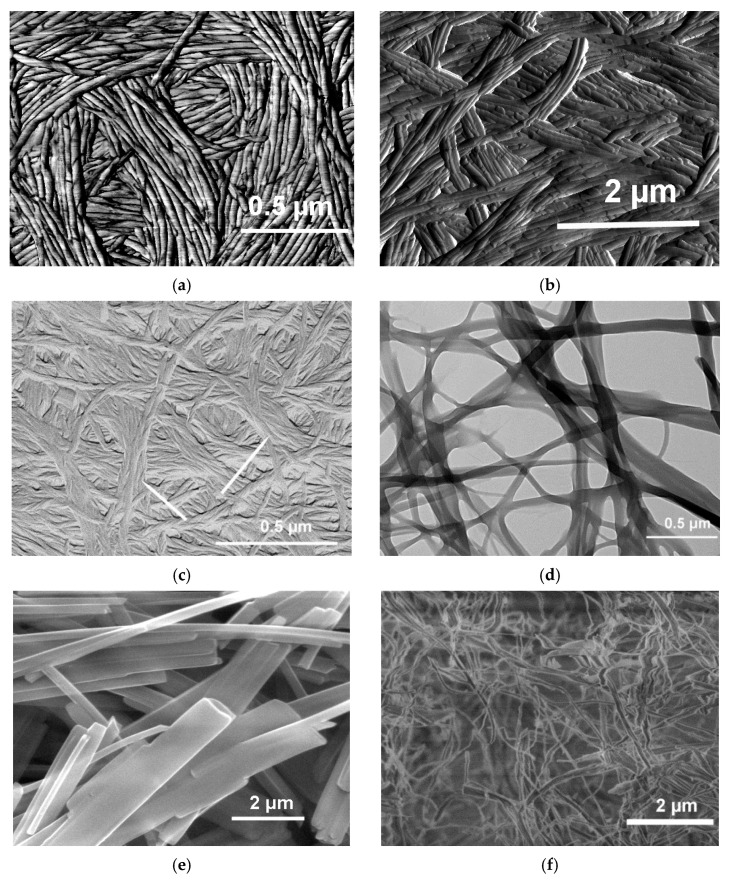
(**a**) AFM micrograph of BHPB-10/*trans*-decahydronaphthalene. Nanotubes arise from the twisting and fusion of lamellae (**b**) AFM micrograph of BHPB-10/cis-decahydronaphthalene. (**c**) SEM micrograph of BHPB-10/*o*-xylene gels, arrows indicate domains where fibrils twist around one another. (**d**) TEM micrograph of OPVR/*trans*-decahydronaphthalene gels. (**e**) SEM Propargyl Ammonium-Based molecule/H_2_O (chemical structure Appendix A Figure A4 (**f**)) SEM TATA/bromobenzene gels. [13,14,33,34].

As stated in the definition section, organogels are made up with an array of fibrillar elements whose mesh size lies in the micrometre range. The gel fibrils display in most cases circular cross-sections (Figure 9a,c), or ribbon-like, rectangular cross-sections (Figure 9b,d). These cross-sections have sizes typically ranging from 100 to 1000 nm.

The morphology depends also upon the solvent type as illustrated by Figure 9a–c. In the case of BHPB-10 the nature of the solvent conformer, *trans* or *cis*-decahydronaphthalene produces different morphologies: nanotubes for the former against lamella for the latter. Similarly, nanotubes of Figure 9c are no longer produced with aromatic solvents.

The pictures shown in Figure 9a,c highlight that fibrils can be connected to one another, through parallel aggregation (Figure 9a), and/or by twisting around one another (Figure 9c), generating in both cases super-fibrils. Connections in ribbon-like structures are generated both by fibril splitting together with parallel aggregation something reminiscent of a railway system (Figure 9d). In other systems, that can be designated as an array of lathes (Figure 9e), a very limited number of connections are established, somehow resembling a Mikado game. Finally, in some systems display a jumble of fibrils (Figure 9f). It goes without saying that the degree of connectedness has a direct bearing upon the rheological properties as will be reviewed below.

**Figure 10:**
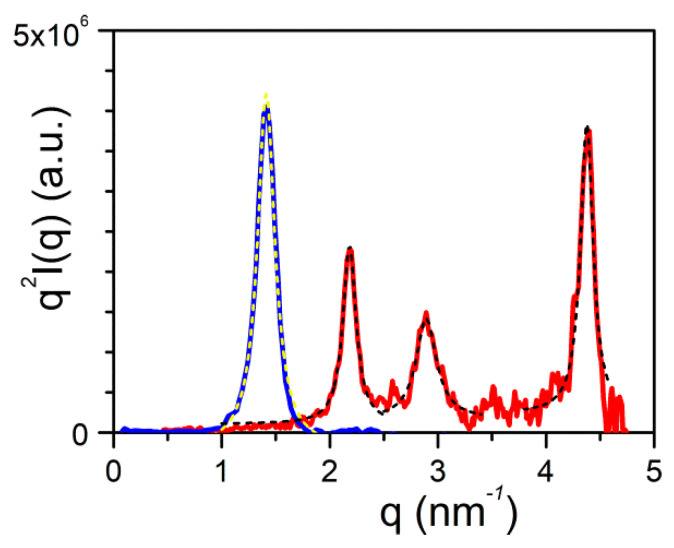
SAXS scattering: blue line is the diffraction patterns for OPVOH (Appendix A Figure A1b), and red line for OPVR (Appendix A Figure A1a) gels in benzyl alcohol [13]. Dashed lines stand for the fits of the different peaks with Equation (16).

**Figure 11:**
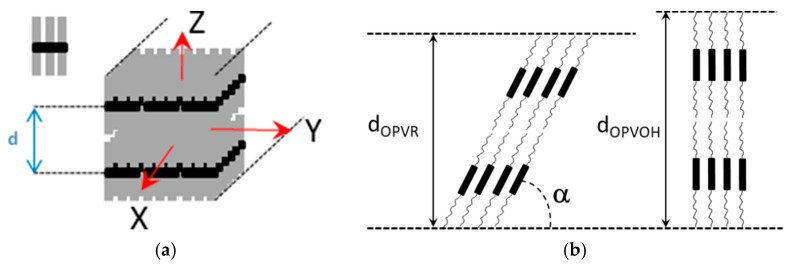
(**a**) Sketch of an OPV fibril, the molecule is shown as a black core with grey aliphatic arms. X is the growth direction (**b**) sketch of the crystal organization seen sideways along the Y-direction. Unlike the OPVOH molecules the OPVR molecules are tilted by an angle α = 41° ± 5°. This gives a core-to-core distance of d = 0.5 nm against d = 0.35 nm for the π-π packing in OPVOH [13].

Here, it is worth emphasizing that a small alteration of the organogelator chemical structure has a dramatic effect on the crystal organization as is illustrated by gels from OPVOH and OPVR in benzyl alcohol (Figure 10). These organogelators only differ in their terminal groups (see Appendix A Figure A1), yet, only one very narrow peak is seen for OPVOH against three peaks for OPVR. This means that the highest order for OPVOH occurs along the z-direction (see Figure 11a), namely, along the 001 crystallographic plane, although the fastest growth rate is along the X-direction, namely, along the 100. Also, Dasgupta et al. have suspected that the benzyl alcohol may interact with the OH group of the OPVOH so as to form a molecular compound along the Y-direction. This may prevent from a long-distance organization in this direction, hence the absence of a 010 crystallographic peak [13]. In addition, the distances between the layer lines determined from electron microscopy diffraction as well as from the SAXS patterns imply that the piling in the X-direction is different. Unlike the OPVOH the OPVR molecules are tilted by an angle α = 41° ± 5° (Figure 11b) [13]. For OPVR the first three peaks are therefore the 001, 010, and 002.

The extent of organization can be estimated from the correlation length *ξ* which is derived from the full width at half maximum (FWHM), Δ*q*, of the diffraction peak through (Figure 10) [35]:

(15) $$\Delta q=\frac{2\pi }{\xi },$$

This means that the smaller Δ*q*, the larger *ξ*, and correspondingly a higher degree of organization.

The FWHM is obtained by fitting the curve with a Lorentzian function:

(16) $$q^{2}I\left(q\right)=S_{B}+\frac{2A}{\pi }\left[\frac{\Delta q}{4{\left(q-q_{o}\right)}^{2}+\Delta q^{2}}\right]$$

where *q_o_* is the position of the peak, *A* is a constant, and *S_B_* is a background signal.

The FWHM of the 001 peak for OPHOH/benzyl alcohol gels gives Δ*q* = 0.2 that is ξ_OPVOH_ = 31.4 nm, against Δ*q* = 0.14 that is ξ_OPVR_ = 45 nm for the 0.01 peak of OPHR/benzyl alcohol gels. Note that the third peak, 002, is probably a second order of the 0.01 peak as Δ*q* is the same.

The molecular structure of the elements responsible for the formation of fibrils can be determined by means of radiation scattering investigations. Techniques such as small-angle X-ray scattering (SAXS) or small-angle scattering neutron (SANS) are now routinely used, and give access to the molecular structure within the range 1–100 nm.

**Figure 12:**
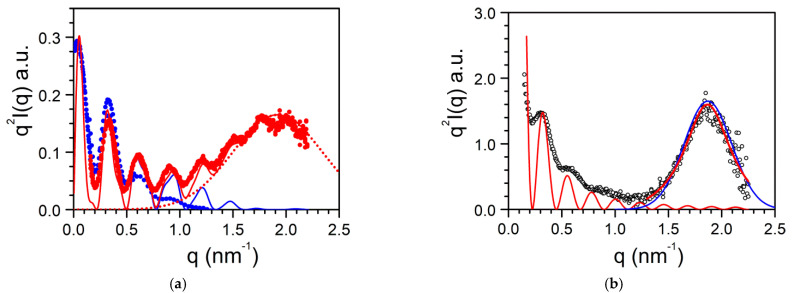
Scattering patterns plotted by means of a Kratky plot (*q*^2^*I*(*q*) vs. *q*): (**a**) Scattering pattern for BHPB-10/*trans*-decahydronaphthalene systems plotted by means of a Kratky plot (*q*^2^*I*(*q*) vs. *q*) for (●) SAXS data; (●) SANS data; blue line fit with Equation (17); dotted line with Equation (19), red line corresponds to relations 17 + 19. (**b**) SAXS data for BHPB-10/*cis*-decahydronaphthalene systems; red line fit with Equation (20); blue line fit of the peak with relation 16 blue line with relation 19 (see text for details) [33].

The case of BHPB-10 is particularly interesting on account of the conspicuous effect on the solvent type on the molecular structure. The gel from BHPB-10/*trans*-decahydronaphthalene gives off a quite rare scattering pattern as it displays several, well-defined oscillations (Figure 12a) [33,36]. These oscillations are not due to Bragg peaks but arise from the cylindrical molecular structures (Figure 9a). Indeed, the fit of the scattering date can be achieved with parallel hollow cylinders whose scattered intensity reads [37]:

(17) $$q^{2}I\left(q\right)=\left[2\pi qC\mu_{L}{\left[\frac{2}{\left(1-\gamma^{2}\right)r_{ext}}\times \left\{J_{1}\left(qr_{ext}\right)-\gamma J_{1}\left(q\gamma r_{ext}\right)\right\}\right]}^{2}\right]\times \overset{n}{\underset{j=1}{\sum }}\overset{n}{\underset{k=1}{\sum }}J_{o}\left(qr_{jk}\right)$$

where the first term in bracket stands for the form factor of hollow cylinders and the second term for the intermolecular interactions. *r_ext_* is the external radius of the cylinder, *γ* the ration of the internal to the external radius, *C* the concentration, *µ_L_* the mass per unit length, and *n* the number of parallel cylinders bunched together. The fit yields *r_ext_* = 12.74 nm, *γ* = 0.74, with a minimum value for *n* equals to 3 yielding Equation (18) [33]:

(18) $$q^{2}I\left(q\right)=\left[2\pi qC\mu_{L}{\left[\frac{2}{\left(1-\gamma^{2}\right)r_{ext}}\times \left\{J_{1}\left(qr_{ext}\right)-\gamma J_{1}\left(q\gamma r_{ext}\right)\right\}\right]}^{2}\right]\times \left[1+2J_{o}\left(2qr_{ext}\right)\right],$$

Using larger n does not improve the fit. The thickness of the nanotubes, e_n_ = 3.3 nm corresponds to the length of the BHPB-10 molecules.

One observes an additional, conspicuous maximum in the X-ray pattern, which peaks at *q* = 1.92 nm^−1^ corresponding to d = 3.27 nm from Bragg’s law. This value, related to the organization within the nanotube, turns out to be close to the length of the BHPB-10 molecule. This maximum can be fitted with a simple Gaussian function:

(19) $$q^{2}I\left(q\right)\sim \;exp-\frac{2.77}{\Delta q^{2}}{\left(q-1.92\right)}^{2}$$

where the full width at half maximum (FWHM) Δ*q* = 0.99.

The maximum seen in SAXS is absent in SANS (Figure 12a). It has been suspected that this arises from a contrast effect together with a solvation of the structure [5]. In neutron scattering experiments the solvent is deuterated while the BHPB-10 contains only hydrogen atoms. If part of the structure includes solvent molecules, then the contrast giving rise to the Bragg peak is likely to vanish. The solvation effect probably explains further why the morphologies differ whether the system is prepared with one or the other conformer of decahydronaphthalene (Figure 9a,b).

Similarly, the SAXS scattering curve for BHPB/*cis*-decahydronaphthalene differs significantly from that obtained in *trans*-decahydronaphthalene (Figure 12b). The scattering curve can be effectively fitted by considering a slab of length *L_c_*, width *l_c_* and thickness *δ_c_*, mass *M* under the conditions *qL_c_* > 1 and *ql_c_* > 1:

(20) $$q^{2}I\left(q\right)=M\frac{8\pi }{L_{c}l_{c}\delta_{c}}\frac{sin^{2}(q\delta_{c}/2)}{q^{2}\delta_{c}}=\frac{8\pi \rho sin^{2}(q\delta_{c}/2)}{q^{2}\delta_{c}}$$

where *ρ* is the slab density. That the oscillations are not so well defined arises from a certain degree of thickness polydispersity. The fit with 20 yields *δ_c_* = 28 nm, a value twice larger than the nanotube external radius.

Here too, a maximum peaks at *q* = 1.87 nm^−1^, namely, d = 3.36. The maximum can be fitted with a Gaussian function (Figure 12a) or a Lorentzian function (Figure 12b). In both cases the FWHM Δ*q* = 0.55 is smaller than the value derived in the case of BHPB-10 in *trans*-decahydronaphthalene. From the value of Δ*q* one derives ξ_cis_ = 11.5 nm and ξ_trans_ = 6.3 nm, values that are consistent with the fact that the lamellar thickness in *cis*-decahydronaphthalene is larger than the nanotube thickness in *trans*-decahydronaphthalene.

**Figure 13:**
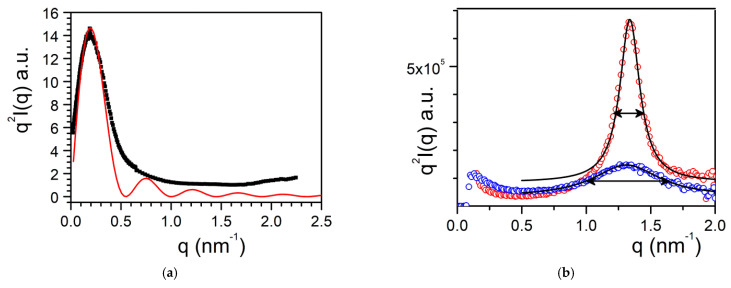
Scattering patterns plotted by means of a Kratky plot (*q*^2^*I*(*q*) vs. *q*) for: (**a**) SANS, BHPB-10/o-xylene; C = 0.01 g/cm^3^; red line fit with relation 21. Note the peak is due to the representation, and is therefore not a Bragg peak [38]. (**b**) Scheme 0. C (◯); C = 0.04 g/cm^3^. Here the peak is a Bragg peak as it is also seen in a I(q) vs. q plot. The curves are fitted with relation 16 and the FWHM, Δ*q*, is shown by double arrows [39].

The effect of the solvent type observed on the morphology of BHPB-10 gels is again emphasized by the intensity scattered by BHPB-10/*o*-xylene gels (Figure 13a), which can be approximately fitted with the structure factor of a solid cylinder:

(21) $$q^{2}I\left(q\right)\sim \pi q\mu_{L}\frac{4J_{1}^{2}\left(qr_{c}\right)}{q^{2}r_{c}^{2}}$$

where *r_c_* = 7 nm. Again, the occurrence of radius polydispersity entails the dumping of the oscillations up to a point where they totally vanish. The value of *r_c_* is noticeably smaller than the external radius measured for the nanotubes. On the basis of this value Khan et al. [38] have considered the occurrence of helices with two BHPB molecules per cross-section instead of nanotubes.

The effect of the quenching temperature on the fibril structure can be determined by measuring the peak broadening corresponding to 001 peak for OPVOH/*cis*-decahydronaphthalene gels. The scattering profile of the 001 peak changes significantly whether the system is quenched at 0 °C or 20 °C (Figure 13b). The FWHM determined from a fit with the Lorentzian function of relation 16 gives Δ*q*(0 °C) = 0.63 with ξ = 10 nm against Δ*q*(20 °C) = 0.19 with ξ = 33 nm. Clearly, the deeper the quench, the thinner the fibrils since this corresponds to the Z-direction (see Figure 11), an effect that chiefly stems from the direct relation between the number of nuclei and the undercooling as mentioned in relation 2.

**Figure 14:**
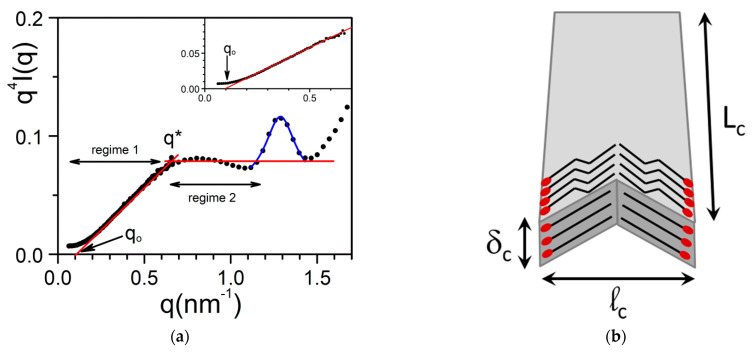
(**a**) Neutron Scattering patterns plotted by means of q^4^I(q) vs. q for propargyl ammonium-based molecule/D_2_O; C= 0.015 g/cm^3^; regime 1 is a fit with relation 23 while regime 2 is the Porod regime. Inset magnification of regime 1; blue line is a fit of the peak with Equation (26). (**b**) arrangement of the molecules in a chevron-like fashion along the length of the lath [34].

In another gel system made up with propargyl ammonium-based molecule/H_2_O, the thickness polydispersity is much larger. The scattered intensity can be fitted by considering a thickness distribution function *w*(*δ*) of the type [34] (Figure 14a):

(22) $$w\left(\delta \right)\sim \;\delta^{-\alpha }$$

with two cutting values for *δ*, namely, *δ_min_*, and *δ_max_*.

For α = 1, one can have two regimes, a transitional regime for *qL_c_* and *ql_c_* >> 1, where *qδ* can take any value:

(23) $$I\left(q\right)=\frac{2\pi \rho }{q^{4}}\left[q\pi -\frac{2}{\delta_{max}}\right]\times \frac{1}{Ln\left(\delta_{max}/\delta_{min}\right)},$$

And for *qδ_min_* >> 1, the intensity reaches the Porod regime [40]:

(24) $$I\left(q\right)=\frac{4\pi \rho }{{\langle \delta \rangle }_{n}q^{4}},$$

where <*δ>_n_* is the number averaged value of *δ_._* which is written:

(25) $$\frac{1}{\delta_{n}}=\left[\frac{1}{\delta_{min}}-\frac{1}{\delta_{max}}\right]\times \frac{1}{Ln\left(\delta_{max}/\delta_{min}\right)},$$

In a *q*^4^*I(q)* representation, one should obtain a straight line in the transitional regime, with an intercept *q_o_* at *I*(*q*)= 0 (inset Figure 14a):

(26) $$q_{o}=\frac{2}{\pi \delta_{max}}$$

and a plateau for *qδ_min_*> 1.

The intercept of the straight lines defining the two regime q* reads:

(27) $$\delta_{min}=\frac{2}{\pi q*}$$

which allows one to determine *δ_min_*. In the present case *δ_max_* = 6.4 nm and *δ_min_* = 1 nm.

It ought to be emphasized that these outcomes from neutron scattering do not mean that a given lath has a constant thickness throughout. A lath may consist of domains with different thicknesses, something which cannot be detected by scattering experiments, particularly if qD > 1, where D is the size of the domains.

Here too, one can observe a Bragg peak for *q* = 1.29 nm^−1^ (d = 4.87 nm) in the low-q range due to the large size of the molecules. As discussed by Morin et al. [34] this can be accounted for by considering a chevron-like structure where molecules are positioned tail-to-tail and head-to-head (Figure 14b). A fit of the peak with a Lorentzian function (relation 16) yields Δ*q* = 0.22 so that ξ = 28.5 nm. One has therefore to contemplate a structure where the molecules lie flat on the *L_c_l_c_* plane to account for the fact that this correlation length is significantly larger than *δ_max_* = 6.4 nm and *δ_min_* = 1 nm.

### 2.4. Rheology

**Figure 15:**
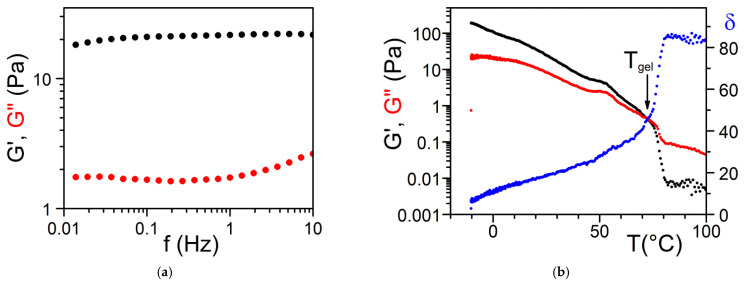
Tri-aryl triamine gels in bromobenzene; (**a**) Variation of *G*’ and *G*” as a function of frequency at T = 20 °C. (**b**) Variation of *G*’, *G*” and *δ* as a function of temperature for a frequency of 1 Hz [28].

The determination of the rheological properties is an essential aspect in the investigations of gels. The goal is to find out whether one is dealing with an ideal gel and/or how far the gel departs from ideality. An ideal network is supposed to possess an elastic modulus E, namely, a direct relation between the applied stress and the deformation *σ* = Ex*ε*, which remains constant either at constant deformation and/or constant stress. Yet, a gel is a special network in that it contains a very large fraction of liquid. As a result, there is always a viscous effect due to the internal frictional force that takes place between adjacent layers of fluid. A gel is therefore best characterized by a complex modulus:

(28) $$G=G^{\prime }+iG^{\prime \prime }$$

where *G*’ is the elastic term (storage modulus) and *G*″ the viscous term (loss modulus). It is usually admitted that a gel of the type organogel [5,12] and/or polymer thermoreversible gel [8,11] can be considered ideal when *G*′ >> *G*″ within the explored frequency range in oscillatory experiments (Figure 15). Another parameter is the angle *δ*, which measures the dephasing between *G*’ and *G*” (tan *δ* = *G*”/*G*’). For a purely elastic system *δ* = 0°, while for a purely viscous system *δ* = 90°.

#### 2.4.1. Origin of Elasticity

The fact that organogels are assemblies of rigid objects should imply that elasticity arises only from fibrils bending. As a result the elastic is of the *enthalpic* type as opposed to the *entropic* type observed in flexible systems such as covalent polymer networks [41]. The theory derived by Jones and Marquès [41] for enthalpic elasticity extended by Guenet to fibrillar gels [11,15] provides a simple relation between the concentration of elastic material, *φ*, and the fractal dimension of the fibril long-axis, *D_f_*:

(29) $$G^{\prime }\sim er_{\sigma }^{4}{\left(\frac{\varphi }{r_{\sigma }^{2}}\right)}^{3+D_{f}/3-D_{f}},$$

where *φ* is the volume fraction of the elastic moiety, *e* is the fibril’s intrinsic modulus, $r_{\sigma }^{2}$ the square of the fibril’s cross-sectional radius (or $r_{\sigma }^{2}=l_{c}\times \delta_{c}$ for rectangular cross-sections).

For straight fibrils *D_f_* = 1 which gives

(30) $$G^{\prime }\sim e\varphi^{2},$$

Note that *φ* can differ from the organogelator concentration because of the possible presence of fibrils not connected to the network that do not participate in the elastic properties (pendent fibrils for instance), or in the case of compounds [15]. As a result, one may obtain an apparent exponent systematically larger than the actual value.

If only deformation of rigid objects is at play, then *G*′ > *G*″ and both must be constant throughout the frequency range. Yet, despite their rigid nature, one cannot exclude the possibility that organogels may experience at least two stress release processes in the linear regime. One process can be the sliding of fibrils onto one another, the second one the breaking/reformation of fibrils, particularly those of thinnest cross-section, and/or those containing defects. This breaking/reformation process may occur when a fibril is forced to cross another for instance. Note that the breaking/reformation process considered here should not be confused with a yield stress.

**Figure 16:**
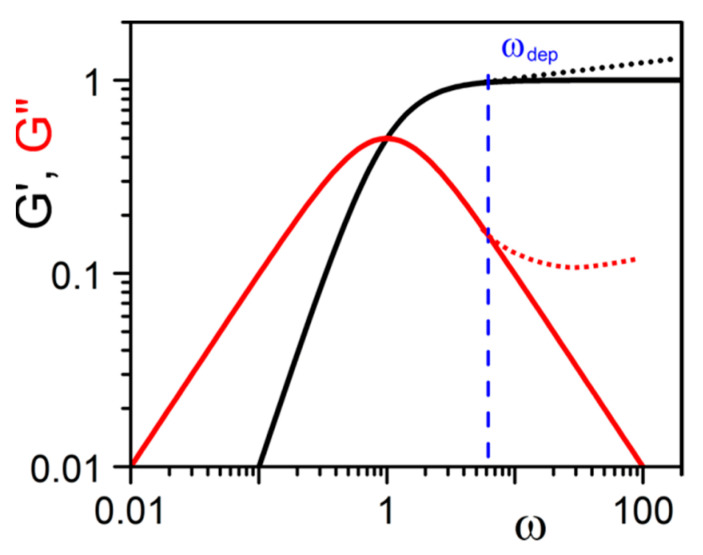
Solid lines represent the theoretical variation of G′ and G″ as a function of frequency from relation 31. Dotted lines above *ω_dep_* stand for the usual experimental departures seen with dynamic polymers for instance [42,43,44].

In this aim, the concepts and theories developed for dynamic polymers by Cates and coworkers [42,43,44] together with the Maxwell model developed for visco-elastic systems are worth contemplating. For oscillatory experiments, *G*′ and *G*″ are written:

(31) $$G^{\prime }=G\frac{\omega^{2}\tau^{2}}{\left(1+\omega^{2}\tau^{2}\right)},\;G^{\prime \prime }=G\frac{\omega \tau }{\left(1+\omega^{2}\tau^{2}\right)}$$

where *τ* is the characteristic time of the system. At low frequencies, *ωτ* < 1, one obtains *G*′ ≈ *ω*^2^ and *G*″ ≈ *ω*. At *G*′ = *G*″ one has *ω* = *τ*^−1^ (Figure 16). Cates has derived *τ* when both processes are competing by combining both characteristic times through:

(32) $$\tau =\left(\tau_{break}\tau_{s}\right),{\;}^{1/2}$$

where *τ_break_* is the time related to the breaking process and *τ_s_* related to the sliding process. If the breaking/reformation process is absent, then the characteristic time is simply *τ* = *τ_s_*_._

It should be kept in mind that this approach is only tentative as the relaxation mechanisms are not precisely known for these types of gels. Evidently, these mechanisms are to differ whether one is dealing with gels displaying a large number of strong junctions (Figure 9d for instance) or gels that are just a jumble of fibrils (Figure 9e,f for instance).

The occurrence of a sliding process has been observed by Collin et al. [12] for a peptide organogel in 1,2,3,4-tétrahydronaphthalene. Although *G*’ is much larger than *G*” in the usual frequency range, the absence of permanent junctions have been evidenced through a series of consecutive deformations. Their experiments consist in applying a given deformation, and then submitting the sample to a vibrating shear mode by means of a piezzorheometer. By compressing further, they have observed that the response remains identical independent of the deformation. To be sure, the compressive deformation has led to an irreversible modification of the organogel. If the gel were not irreversibly deformed, then the stress should have increased with increasing the deformation.

This type of behaviour has already been reported by Guenet and McKenna for polymer thermoreversible gels [9]. That *G*′ > *G*″ in the usual frequency range is therefore not an absolute criterion for defining a true gel as already discussed above. Experiments reported by Lescanne et al. [45] by exploring lower frequencies on propylene carbonate gels prepared in 2,3-di-n-decyloxyanthracene are rather reminiscent of the behaviour shown in Figure 16. Similarly, results by Terech et al. on 12-Hydroxystearic Acid/dodecane gels [46] suggest that *G*″ increase while *G*′ decreases in the low frequency range for *ω* < *ω_dep_* which suggest the type of behaviour of Figure 16. These observations highlight departure from the ideal gel behaviour, the only difference with covalent polymer and/or dynamic polymer solutions being a characteristic time *τ* much larger. The data from Lescanne et al. [45] or Terech et al. [46] suggest that this time *τ* could be 10 or even 1000 times larger.

Since very low frequencies are usually not accessible with the available rheometers, the only way to find out whether the relaxation time is finite consists in performing relaxation experiments, namely, applying a deformation and measuring the resulting stress. If the system is a true gel, then the relaxation rate dσ/dt should be close to zero. This approach has been used by Guenet and McKenna, which allowed them to find out that some polymer thermoreversible gels display significant relaxation rates in spite of G’>G” in the usual frequency range [9].

#### 2.4.2. The Gel Point: Onset Gelation Concentration

In the seventies the percolation model has been developed for describing the gelation mechanism of chemical networks [47,48]. Basically, reactants, monomers or polymer chains, are gradually cross-linked up to the point of generating an aggregate of “infinite” size. The degree of the cross-linking reaction p, namely, the fraction of connected objects, increases up to a value p_c_, designated as the *percolation threshold*. The value of p_c_ is independent of the size of the vessel where the cross-linking process is carried out. In this sense p_c_ is a critical parameter.

In the case of physical gels, that is organogels and polymer thermoreversible gels, use of the percolation model has also been contemplated. Another model, named fibrillary model, has also been put forward [49]. For using the percolation model, it has been assumed that the equivalent of the gelation threshold should be the critical concentration *C_gel_*, namely, the concentration at which a gel is formed. Can we really consider *C_gel_* as equivalent to p_c_? In physics, a critical phenomenon is related to the occurrence of critical points. Most of them are characterized by the divergence of some correlation length. Critical phenomena are usually phase transitions of second order, and are described by critical exponents, universality, fractal behaviour, and ergodicity breaking. It is actually true that the divergence of the size of the aggregate is supposed to occur at *C_gel_*. So, it is tempting to use *C-C_gel_* as equivalent to p-p_c_.

**Figure 17:**
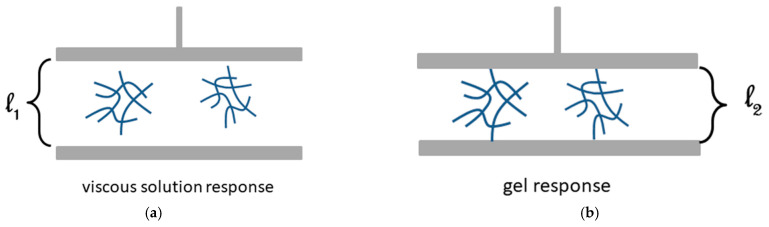
Schematic representation of a plate-plate set-up in a rheometer: (**a**) aggregates of size smaller than *l*_1_ giving a viscous response. (**b**) aggregates of size larger than *l*_2_ giving a gel response [50].

Yet, *C_gel_* depends on the size of the measuring device as illustrated in Figure 17. If the size of the aggregate is smaller than the plate-plate distance in the rheometer, then a viscous response will be observed (Figure 17a). Now, if the size of the aggregate is larger than this distance, then a gel response will be seen (Figure 17b) [50]. This so because the aggregates are small pieces of gel unlike the objects formed below p_c_ in the percolation model.

It is worth estimating the gelation concentration before elaborating further on its non-criticality. Here, the calculation of *C_ge_*_l_ is carried out in the same way as the overlap concentration C* is calculated for polymer solutions. It is simply the mass of one fibril divided by the volume of the sphere within which this fibril is inscribed. *C_gel_* reads accordingly [15,51]:

(33) $$C_{gel}=\frac{6M}{\pi S_{F}^{3}}$$

where *S_F_* is the end-to-end distance of the fibril’s long axis. Introducing the contour length of the fibril *S_F_* ~ $L_{F}^{1/D_{F}}$ where *D_F_* is the fractal dimension of the fibril’s long axis (for a straight fibril *S_F_* = *L_F_*).

(34) $$C_{gel}\sim \frac{6\rho r^{2}}{L_{F}^{\left(\frac{3-D_{F}}{D_{F}}\right)}}$$

where *ρ* is the fibril’s density, and *r* the fibril’s cross-section.

This implies that *C_gel_* depends upon the fibril’s cross-section, which in turn depends on the quenching temperature as mentioned in the previous section. If the number of fibrils is kept constant, then decreasing r entails an increase of *L_F_*, which results in a significant decrease of *C_gel_*. Alternatively, if *L_F_* is kept constant, then decreasing *r* entails an increase of the number of fibrils, and correspondingly a decrease of *C_gel_*.

This considerations therefore entail that for a given organogelator concentration C, *C-C_gel_* is not a relevant parameter although frequently considered [52,53]. Indeed, *C_gel_* may also vary with the investigation temperature since the gel fraction determined by relation 10 may vary drastically depending upon the shape of the phase diagram.

A critical parameter is not supposed to depend upon the path followed to reach a particular state of the system. Then, *C_gel_* cannot be considered a critical parameter. A term such as “*onset gelation concentration*” seems probably more appropriate for designating the gel point. Also, it should be clear that organogelation, and more generally thermoreversible gelation involving first order transitions, is not a percolation process.

#### 2.4.3. Modulus vs. T-C Phase Diagram

One can establish a direct relation between the gel fraction and the modulus from the T-C phase diagram. In the simplest case described in Figure 4a the liquidus line is a function of the organogelator concentration *T* = *f*(*C_org_*). Therefore *C_org_* = *f*^−1^(*T*) where *f*^−1^ is the inverse function of *f*. If one assumes for the sake of simplicity that *φ_rich_* = 1, then one obtains [5]:

(35) $$\varphi_{gel}=\frac{C_{org}-f^{-1}\left(T\right)}{1-f^{-1}\left(T\right)}$$

where *φ_gel_* is the gel fraction.

**Figure 18:**
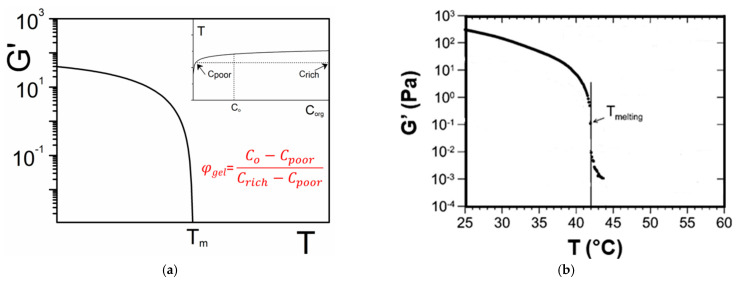
(**a**) theoretical variation of the elastic modulus as a function of temperature. *φ_gel_* is the gel volume fraction calculated as shown. Inset: the corresponding liquidus used for the calculation of *G*′. (**b**) experimental results obtained by Collin et al. on a peptide-based organogelator in 1,2,3,4-tétrahydronaphthalene Appendix A Figure A5 [12].

An example is shown in Figure 18a where an arbitrary function of the type T~ [Log(a*C_org_)]^1/2^ is used to mimic the liquidus line (inset of Figure 18a). The inverse function is then *C_org_*~ a^−1^exp(T^2^). If one further assumes that the elastic modulus varies as:

(36) $$G^{\prime }\sim \;\varphi_{org}^{2}$$

one obtains the variation of *G*′ vs. T shown in Figure 18a. Experimental results obtained by Collin et al. on a peptide-based organogelator in 1,2,3,4-tétrahydronaphthalene (Figure 18b) do exhibit this type of behaviour [12]. Clearly, mapping out the T-C phase diagram is an essential step for a better understanding of the rheological properties of a gel as a function of temperature.

## 3. Conclusions

This short paper advocates the extensive study of the physical properties of organogel in order to throw some light on the gel formation mechanism, and ultimately to be able to estimate the probability for a given molecule to produce a gel. Several attempts in this aim have been already put forward [54,55].

Sticking to the definition developed above, namely, a gel must consist of highly elongated, crystalline objects, implies to design systems that are to display a crystallization behaviour that privileges crystal growth in one direction. As suggested by Guenet the candidates must be “*chimeras*” molecules, that is to be an assembly of parts with differing interaction properties such as hydrogen bonding, Van der Waals interaction or/and π-π interaction. The presence of long aliphatic arms is often a prerequisite [5]. The OPVOH molecules synthesized by Ajayaghosh and coworkers stand as a paradigm in this respect [4] (Appendix A Figure A1b).

The above conditions automatically entails that these potentially gelling molecules possess rather large molecular weights. They are often designated as low-molecular weight gelator (LMWG) as these gelators usually possess molecular weight larger than usual organic molecules, such as solvents, but much smaller molecular weights than macromolecules. Yet the term “low-molecular-weight “is confusing, after all solvents are also low-molecular weight molecules! It is felt that the term “*mesomolecules*” might be more appropriate for naming these types of molecules.

These prerequisites are chiefly indicative as the gelation behavior can be totally different in a series of molecules. For instance, the capability of BHPB molecules to produce nanotubes depends strongly upon the length of the aliphatic arms [56], which makes uncertain prediction of its gelling property. Other major factors to be considered are the path followed when cooling the solution, especially when a miscibility gap exists, the solvent type, and the like. For instance, BHPB forms nanotubes in aliphatic solvents but not in aryl solvents [38], which suggests the possible existence of molecular compounds [5].

## Figures and Tables

**Figure A2:**
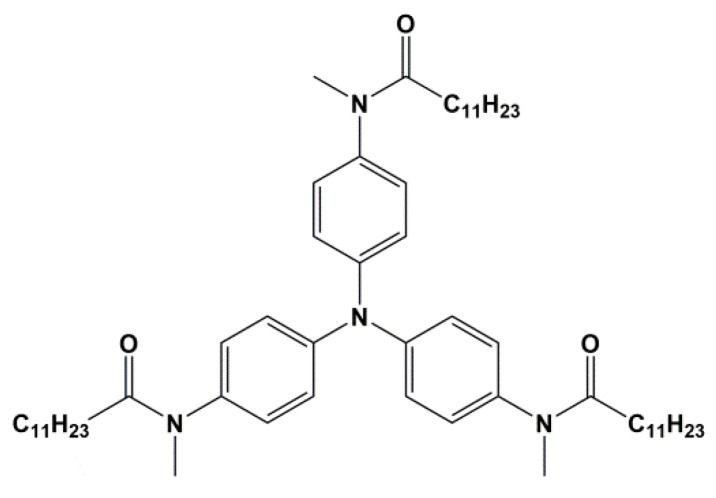
TATA: tri-aryl tri-amine. For synthesis see reference [27].

**Figure A3:**
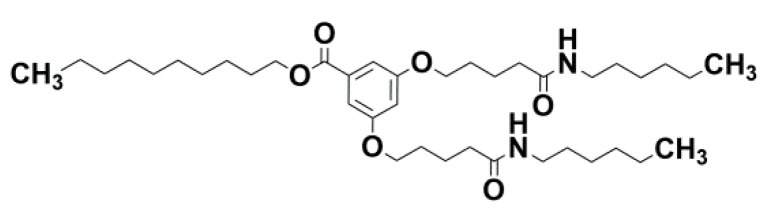
BHPB-10; number 10 stands for the number of carbons in the aliphatic arm. For synthesis see reference [36].

**Figure A4:**
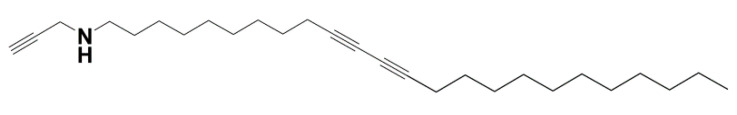
*N*-Methyl-*N*-(pentacosa-10,12-diyn)-propargylamine. For synthesis see reference [27].

**Figure A5:**
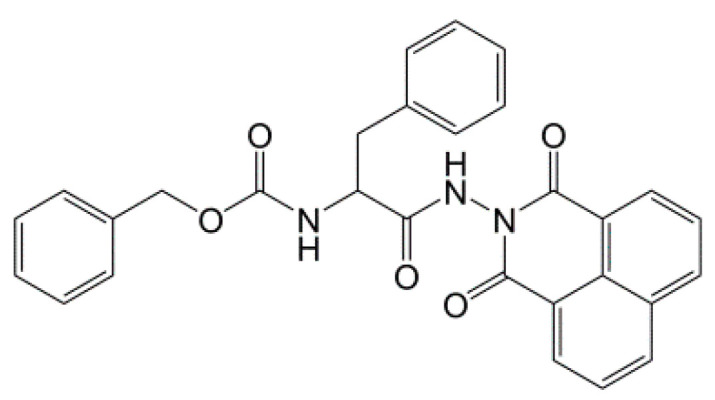
Peptide protected by a benzyloxycarbonyl group at its N-terminal, and by a hydrazine naphthalimide at the C-terminal [12].

## Data Availability

Data available on request.

## Appendix A

Chemical structures of the molecules described in the article.
